# Extracting expression modules from perturbational gene expression compendia

**DOI:** 10.1186/1752-0509-2-33

**Published:** 2008-04-10

**Authors:** Steven Maere, Patrick Van Dijck, Martin Kuiper

**Affiliations:** 1 Department of Plant Systems Biology, VIB, Technologiepark 927, B-9052 Ghent, Belgium; 2 Department of Molecular Genetics, Ghent University, Technologiepark 927, B-9052 Ghent, Belgium; 3 Department of Molecular Microbiology, VIB, Kasteelpark Arenberg 31, B-3001 Leuven, Belgium; 4 Laboratory of Molecular Cell Biology, Katholieke Universiteit Leuven, Kasteelpark Arenberg 31, B-3001 Leuven, Belgium

## Abstract

**Background:**

Compendia of gene expression profiles under chemical and genetic perturbations constitute an invaluable resource from a systems biology perspective. However, the perturbational nature of such data imposes specific challenges on the computational methods used to analyze them. In particular, traditional clustering algorithms have difficulties in handling one of the prominent features of perturbational compendia, namely partial coexpression relationships between genes. Biclustering methods on the other hand are specifically designed to capture such partial coexpression patterns, but they show a variety of other drawbacks. For instance, some biclustering methods are less suited to identify overlapping biclusters, while others generate highly redundant biclusters. Also, none of the existing biclustering tools takes advantage of the staple of perturbational expression data analysis: the identification of differentially expressed genes.

**Results:**

We introduce a novel method, called ENIGMA, that addresses some of these issues. ENIGMA leverages differential expression analysis results to extract expression modules from perturbational gene expression data. The core parameters of the ENIGMA clustering procedure are automatically optimized to reduce the redundancy between modules. In contrast to the biclusters produced by most other methods, ENIGMA modules may show internal substructure, i.e. subsets of genes with distinct but significantly related expression patterns. The grouping of these (often functionally) related patterns in one module greatly aids in the biological interpretation of the data. We show that ENIGMA outperforms other methods on artificial datasets, using a quality criterion that, unlike other criteria, can be used for algorithms that generate overlapping clusters and that can be modified to take redundancy between clusters into account. Finally, we apply ENIGMA to the Rosetta compendium of expression profiles for *Saccharomyces cerevisiae *and we analyze one pheromone response-related module in more detail, demonstrating the potential of ENIGMA to generate detailed predictions.

**Conclusion:**

It is increasingly recognized that perturbational expression compendia are essential to identify the gene networks underlying cellular function, and efforts to build these for different organisms are currently underway. We show that ENIGMA constitutes a valuable addition to the repertoire of methods to analyze such data.

## Background

Over the last decade, the availability of fully sequenced genomes and the development of high-throughput technologies such as DNA microarray-based transcript profiling have fuelled an exponential increase in the volume of functional genomics data. This has led to a renewed interest in the study of molecular biology at the system level [1-3].

The central paradigm in systems theory is that one can learn about a system by perturbing it and measuring the response. This principle also applies to biological systems. Since mRNA levels can nowadays easily be measured on a genome-wide scale, expression profiling has become a first method of choice for assessing the molecular response to experimental perturbation (the molecular phenotype). Considerable efforts are put into creating compendia of expression profiles under genetic, chemical or environmental perturbations [4-6] or in different tissues [5,7,8]. Such data compendia basically constitute a series of snapshots of expression states under a variety of conditions, and they contain a wealth of information concerning the underlying transcriptional network structure of an organism. However, devising methods to efficiently and reliably extract that information is still a challenging task.

Clustering of gene expression data allows the inference of functional correlations between genes through what was dubbed the 'guilt-by-association' principle [9]. A classical clustering process consists of two steps [10]. First, a matrix of distances between expression profiles is calculated using a distance or similarity measure, such as Pearson's centered correlation coefficient (PCC). Based on this distance matrix, the actual clustering algorithm, for instance average linkage hierarchical clustering, groups similar profiles together. Traditional clustering methods are well suited for analyzing time-series expression data, but they fall short when applied to perturbational data, because the underlying similarity measures, such as PCC, primarily capture global correlation tendencies. However, in compendia of perturbed expression profiles, genes do not necessarily show similar behavior under all experimental conditions: they may be coexpressed under some conditions and follow different expression regimes under other conditions. One of the consequences is that genes may be coexpressed with multiple expression modules depending on the conditions, or in other words, expression modules may overlap.

These observations stimulated the development of alternative clustering strategies. The process of detecting subsets of genes that exhibit similar expression behavior across a subset of conditions is known as biclustering. Several biclustering strategies exist today, each using its own heuristic approach to tackle this complex problem ([11] and references therein). Some biclustering methods use a greedy iterative search strategy to uncover biclusters, progressively subdividing, or adding and removing rows and columns from the biclusters obtained in a previous iteration in order to maximize a local score function [12-15]. Others use linear algebra [16] or adopt a graph-theoretic approach to biclustering [17,18]. Yet other methods identify biclusters by proposing a statistical model and estimating the distribution parameters that minimize a certain model fit criterion [19-25]. A feature that most biclustering methods share is that they do not explicitly define similarity measures on the global space of expression profiles, but rely on the emergent properties of groups of genes and conditions in order to identify significant subpatterns in the data.

Evidently, a wide variety of biclustering algorithms exist, each of them having their own strengths and weaknesses. For example, some of these methods are intrinsically less suited to find overlap between biclusters because they mask previously found biclusters with random noise [12,22], or because they partition the data [16,21,24]. Others require extensive parameter tweaking, require the user to specify the desired number of biclusters in advance or generate very small or large (amounts of) biclusters or highly redundant biclusters (see e.g. comparison in [23]). Some have no publicly available implementation or are rather cumbersome to use, and most of them, notable exceptions being SAMBA/EXPANDER [17,26], Genomica [21] and cMonkey [23], do not integrate or overlay other types of biological data, hampering their use as biological discovery tools.

Also, to our knowledge, none of the existing biclustering methods uses the variational information in replicated expression experiments. This information is routinely and successfully used to detect genes that are differentially expressed under a given perturbation [27]. The main reason why biclustering methods do not use differential expression information is that they do not specifically focus on the analysis of perturbational data. Discretization-based biclustering methods such as SAMBA [17] and BiMax [18] could probably easily be modified to assess up- and downregulation of gene expression based on *p*-values for differential expression. In their current implementation, however, these methods use rather arbitrary log-ratio or percentage cutoffs for this purpose.

In this study, we present a novel method, called ENIGMA, that addresses some of these issues. Our goal was to build a method that: (i) leverages differential expression analysis results to extract co-differential expression networks and expression modules from perturbational gene expression data, (ii) is able to detect significant partial coexpression relationships between genes and overlap between modules, (iii) depends on parameters that can be automatically optimized or set on reasonably objective grounds. (iv) produces a realistic amount of modules, and (v) visually integrates the expression modules with other data types such as Gene Ontology (GO) information [28], transcription factor (TF) binding data, protein and genetic interactions, in order to facilitate the biological interpretation of the results. Below, we outline the ENIGMA algorithm, test our methodology on artificial expression data and compare its performance to other methods. We also apply ENIGMA to a perturbational microarray compendium for budding yeast [4] in order to assess its potential to generate testable hypotheses on real biological data.

## Results

### Algorithm

**Figure 1:**
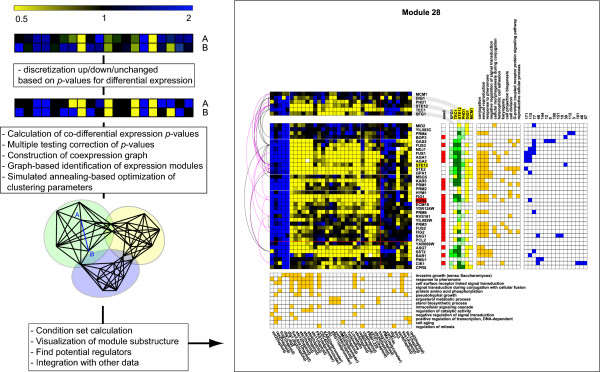
**Global methodology overview**. To the right is a figure of module 28, a module enriched in mating-related genes learned from the Rosetta dataset [4]. See Figure 4 for interpretation guidelines.

A global overview of the methodology is given in Figure 1. Briefly, ENIGMA takes as input a set of perturbational expression data, externally calculated *p*-values for differential expression (e.g. using the limma package in Bioconductor [29]) and other data types if available. ENIGMA uses a novel combinatorial statistic to assess which pairs of genes are significantly co-differentially expressed (henceforth abbreviated as coexpressed for the purpose of readability). The resulting coexpression *p*-values are corrected for multiple testing and translated to edges in a coexpression network, which is clustered into expression modules (i.e. groups of significantly co-differentially expressed genes) using a graph-based clustering algorithm inspired on the MCODE algorithm [30]. The clustering procedure depends on two parameters that control the density of individual modules and the overlap between modules. The main reason why we chose a two-tier clustering approach (data → coexpression network → clustering) is that it allows simulated annealing-based optimization of the clustering parameters to obtain optimal coverage of the coexpression network, in terms of module overlap and redundancy. The graph clustering method we use is very fast, which allows the parameters to be optimized in a reasonable amount of time. In the postprocessing phase, ENIGMA determines relevant condition sets for each module, visualizes their substructure and overlap with other modules, screens the modules for enriched GO categories, suggests potential regulators for the modules based on regulator-module coexpression links and enrichment of TF binding sites, and overlays protein and genetic interaction data.

#### Combinatorial statistic

Consider the expression profiles of two genes *A *and *B *under *N *perturbations (see Figure 1). Each gene is represented by a profile of *N *fields. The gene expression values are discretized into three categories (upregulated, downregulated, unchanged) based on their differential expression *p*-value. If the gene is significantly upregulated in a given experiment (by default if *p *< 0.01), the corresponding field is labeled blue. Experiments in which the gene is significantly downregulated are similarly labeled yellow, and the remaining fields are labeled black. Let us now assume that the profiles of *A *and *B *contain *a*_*x *_and *b*_*x *_blue fields respectively, as well as *a*_*y *_and *b*_*y *_yellow fields, and that they have *x *blue and *y *yellow fields in common. We want to assess whether this overlap is statistically significant. If the response of the genes *A *and *B *to the perturbations were uncorrelated (null hypothesis), the blue and yellow fields would be independently distributed on both profiles. Under this hypothesis, the probability that the profiles overlap on exactly *x *blue and *y *yellow positions is given by the following recursive formula:

(1) $$P(x,y)=\frac{\left(\begin{array}{c} a_{x}\\ x \end{array}\right)\left(\begin{array}{c} a_{y}\\ y \end{array}\right)\left(\begin{array}{c} N-x-y\\ b_{x}-x \end{array}\right)\left(\begin{array}{c} N-b_{x}-y\\ b_{y}-y \end{array}\right)}{\left(\begin{array}{c} N\\ b_{x} \end{array}\right)\left(\begin{array}{c} N-b_{x}\\ b_{y} \end{array}\right)}-\underset{\left(x^{\prime },y^{\prime })\neq (x,y\right)}{\sum_{x^{\prime }=x}^{\min(a_{x},b_{x})}\sum_{y^{\prime }=y}^{\min(a_{y},b_{y})}\left(\begin{array}{c} x^{\prime }\\ x \end{array}\right)\left(\begin{array}{c} y^{\prime }\\ y \end{array}\right)}P(x^{\prime },y^{\prime })$$

The probability of observing an overlap of at least *x *blue and *y *yellow fields by chance is then expressed by the cumulative distribution:

(2) $$P_{c}(x,y)=\sum_{x^{\prime }=x}^{\min(a_{x},b_{x})}\sum_{y^{\prime }=y}^{\min(a_{y},b_{y})}P(x^{\prime },y^{\prime })$$

Equation 1 can be understood by assuming that profile *A *is given, and that we randomly distribute *b*_*x *_blue and *b*_*y *_yellow positions on profile *B*. The denominator of the first term then represents the total number of possible profiles *B*. The numerator represents the combinations in which *x *blue and *y *yellow matching positions are selected, and the residual positions are chosen at random. However, in this manner, a number of combinations are selected while having more than exactly *x *blue and/or *y *yellow matching positions.

Moreover, combinations with *x' *> *x *blue and/or *y' *> *y *yellow matching positions are counted C(*x'*, *x*)·C(*y'*, *y*) times, hence the second term (see Additional file 1).

Although the probabilistic question formulated above can be cast in terms of contingency tables, the hypothesis tested by our statistic is different from that tested by standard contingency table analysis methods such as the *χ*^2 ^test. For example, situations in which a large amount of blue (upregulated) fields in profile *A *are perfectly mapped onto the black fields (up nor down) in profile *B *would yield a significant *χ*^2 ^*p*-value, whereas they would not yield a significant *p*-value using equation 2. Our statistic only considers mappings of up- and down-regulation of the expression of a gene to up- or down-regulation of another gene to be meaningful for assessing coregulation, a premise which is motivated by the perturbational nature of the data we aim to analyze. Black fields are considered less informative from the perspective of coregulation.

#### Multiple testing correction of coexpression p-values

In our probabilistic setup, each comparison of two profiles can be considered an individual test. For *N *genes, *N*(*N *- 1)/2 tests are performed to fish for co-differential expression relationships. Consequently, the obtained *p*-values have to be adjusted in order to control the type I error rate. The raw *p*-values are corrected for multiple testing with the Benjamini & Hochberg procedure, which controls the False Discovery Rate (FDR) [31].

#### Graph-based clustering

The set of significant coexpression relationships at a certain FDR threshold (by default FDR = 0.05) is translated to a network, with nodes and edges representing genes and significant coexpression relationships, respectively. ENIGMA identifies coexpression modules from this network using a graph clustering technique inspired by the MCODE algorithm [30]. To identify potential module seeds, all nodes *v *are weighted based on the density of the highest *k*-core of the node neighborhood *N*_*v*_, denoted as the *k*_*max*_-core of *v *(a *k*-core of a graph is a maximal subgraph in which each node has at least degree *k*). Analogous to Bader and Hogue [30], the core-clustering coefficient *C*_*core*,*v *_is defined as the density of the *k*_*max*_-core of *v*, and the weight *w*_*v *_= *C*_*core*,*v*_·*k*_*max*,*v*_.

The *k*_*max*_-core of the node with the highest weight is taken as the first module seed. This module seed then grows by accreting nodes on which it exerts a pull above a certain threshold *ν*_2_. The pull of a module with seed *S *on a node *v *outside the module is defined as |*N*_*v *_∩ *S*|/|*S*|. The next module is then initiated by taking the *k*_*max*_-core of the node with the highest weight in the remaining graph. An additional constraint is set by requiring that the overlap between the new seed *S *and any existing module *M *does not exceed *ν*_1_·min(|*S*|,|*M*|). While the threshold *ν*_2 _controls the size and density of individual modules, *ν*_1 _controls the spacing or overlap between modules. Both parameters are optimized automatically.

#### Clustering parameter optimization

In order to optimize the clustering parameters, the quality of the clustering for a given (*ν*_1_, *ν*_2_) is assessed by comparing the known input coexpression network (i.e. the network obtained in the first phase of the ENIGMA algorithm) with the output coexpression network inferred by the modules. The latter is constructed by translating the modules to fully connected components in the output network (see Additional file 1 Figure S1 A). If we consider true/false positives (*tp *resp. *fp*) to be coexpression edges inferred by the clustering that are present/absent in the input coexpression network, and false negatives (*fn*) as edges present in the input network that are not inferred by the clustering, we can define the precision *P' *= *tp*/(*tp *+ *fp*) and the recall *R' *= *tp*/(*tp *+ *fn*) of the clustering result. ENIGMA uses the *F'*-measure, i.e. the harmonic mean of recall (*R'*) and precision (*P'*), *F' *= 2*P'R'*/(*P' *+ *R'*), as a measure for the quality of the clustering. We use the notation *P'*, *R'*, *F' *instead of the more commonly used *P*, *R*, *F *in order to distinguish between two different flavours of the *F*-measure used in this study for different purposes. In contrast to the regular *F*-measure (Additional file 1 Figure S1 C), the *F'*-measure penalizes overpredicted edges in order to avoid unnecessary overlap between the expression modules: an edge (*A*, *B*) that is inferred multiple times from the clustering, because the genes *A *and *B *belongs to the intersection of multiple (say *x*) modules, is counted as 1 *tp *and *x *- 1 *fp*. This is equivalent to drawing *x *edges between the genes *A *and *B *in the output coexpression network. Since there is only one edge in the input network, the *x *- 1 remaining edges can be considered false. This penalization strategy has the intuitively pleasing property of not affecting the recall, but lowering the precision of the clustering result when the amount of edges 'explained' by multiple modules increases.

The parameters *ν*_1 _and *ν*_2 _are now optimized by Monte-Carlo Simulated Annealing (MCSA) [32,33] using *F' *as the optimization criterion. Starting from an random initial guess for the parameters (*ν*_1_, *ν*_2_), random steps are taken in parameter space. A step is accepted if

(3) rand(1) <*e*^Δ*F'*/*T*^

with rand(1) a random number drawn uniformly from the interval [0,1], Δ*F' *the change in *F'*-measure and *T *the simulated annealing parameter or 'temperature', which gradually decreases during the course of the optimization according to an exponential scheme *T*_*i *_= *r*_*c*_*T*_*i*-1_, with *r*_*c *_the cooling rate. ENIGMA uses a two-stage MCSA procedure. In the first stage, a rough MCSA search of the clustering parameter space is performed in order to identify the most interesting parameter region (default MCSA settings: *T*_begin _= 0.1, *T*_end _= 0.001, *r*_*c *_= 0.99, parameter step size = 0.05). In the second stage, a finer MCSA search is performed starting from the optimum obtained in the first stage (default MCSA settings: *T*_begin _= 0.01, *T*_end _= 0.0001, *r*_*c *_= 0.995, parameter step size = 0.01). At the end of each stage, an additional gradient descent is performed toward the nearest local optimum of *F'*. By default, ENIGMA performs 3 MCSA runs, starting from randomly chosen (*ν*_1_, *ν*_2_). The convergence of the solutions of multiple runs can be used as a check on the adequacy of the MCSA parameter settings.

#### Postprocessing of modules

For each gene module, ENIGMA determines a condition set by selecting those conditions that show enrichment of up- or downregulated genes in the module (hypergeometric test, default FDR = 0.05). Thus, for a given module, the condition set contains the experimental conditions that elicit a significant and specific response in the module (as compared to the overall response) and, by consequence, have been most influential in shaping the module. The resulting 'bicluster' does not necessarily have a uniform expression pattern over all genes, but may show subpatterns for some genes under certain conditions, possibly indicating involvement in other expression modules. These subpatterns are visualized by hierarchically clustering the module's expression data in both dimensions, using the cosine correlation coefficient (cos*θ*) as a similarity measure. The clustering tree can optionally be separated into leafs to make the subdivision more clear (default threshold cos*θ *= 0.65). Although conditions that show differential patterning within one module might appear to be irrelevant for the module as a whole, they are important for at least part of the module and may provide insight into inter-module connections or further substructure within the module.

In an attempt to provide the user with clues on how the expression modules are regulated, ENIGMA searches for 'regulators' that are significantly more connected to a module, through positive or negative coexpression edges, than expected at random (hypergeometric test, default FDR = 0.05). Potential regulators are selected from a user-defined list or a user-defined set of GO classes. When chromatin immunoprecipitation (ChIP) or TF motif data are available, ENIGMA also screens the modules for enriched TF binding sites (hypergeometric test, default FDR = 0.05). The expression profiles of significantly coexpressed or binding regulators are visualized on top of the modules. Significantly enriched GO terms for both the gene and condition sets of the modules are determined using the BiNGO [34] software, which is incorporated in ENIGMA (hypergeometric test, default FDR = 0.05). Finally, ENIGMA visually maps the available protein interaction data and genetic interaction data on the modules.

### Testing on artificial data

#### Generating artificial expression data

To assess the performance of our method and compare ENIGMA to other methods, we performed tests on artificial gene expression data. We generated two types of artificial expression data, namely expression data containing overlapping biclusters (modular data) and expression data containing partially coexpressed genes but no biclusters (non-modular data). In both cases, we built 10 expression datasets of 1000 genes by 100 experiments (in log_2 _ratio format). For each dataset, artificial background expression data were randomly sampled from a normal distribution with mean *μ *= 0 and variance *σ*^2 ^= 0.16. For the modular datasets, we implanted 20 biclusters in this background, each encompassing between 1–5% of all genes and 10–50% of all conditions. Bicluster sizes, member genes and conditions are chosen at random, with the restriction that at most 30% of the genes and 50% of the conditions overlap between any 2 biclusters (percentages relative to the smallest of the 2 biclusters). Except for a noise component (see further), all genes in a bicluster share the same expression profile over the bicluster conditions. However, a bicluster can be partially overwritten by other biclusters. The bicluster profiles are sampled from a bimodal distribution consisting of 2 normal modes with means *μ*_1 _= -1 (for down-regulated expression) and *μ*_2 _= 1 (for up-regulated expression) and variances $\sigma_{1}^{2}=\sigma_{2}^{2}=0.49$. The expression profiles of individual genes in a bicluster are noisified by adding normally distributed noise (*μ*_*n *_= 0 and *σ*_*n *_= 0.2|*x*| with |*x*| the amplitude of the log ratio expression of the gene in the given condition). The variances, bicluster size and overlap parameters are chosen so that the overall distribution of the simulated log ratio expression values mimicks the distribution of log ratio expression values in the Rosetta compendium [4] up to a scale factor (see Additional file 1 Figure S2). Note that, apart from the distribution of expression ratios, the structure of these toy datasets does not necessarily bear any resemblance to real biological data.

For the non-modular datasets, we implanted 500 pairs of partially coexpressed genes (co-differentially expressed under 10–50% of all conditions) in the background. The expression profiles are constructed as described above. The resulting expression value distribution again mimicks the Rosetta distribution (see Additional file 1 Figure S2).

Unlike for real data (see below), we used log_2 _ratio thresholds to discretize the expression values of the artificial datasets, because the generation of meaningful artificial differential expression *p*-values proved to merit further study in its own right. Therefore, the artificial data cannot be used to assess the advantage of including variational information in ENIGMA's discretization step (instead, we performed a qualitative comparison of *p*-value and log-ratio based discretization on real data, see below). On the other hand, we can still compare the performance of ENIGMA with other methods that do not use variational information. We used a log_2 _ratio threshold of 1 for upregulation and -1 for downregulation, corresponding to the means of the distributions used to generate the bicluster profiles. In other words, half of the datapoints in the biclusters are presumed not to be significantly over- or underexpressed.

#### Performance of ENIGMA on artificial data and comparison with other methods

The performance of ENIGMA on these toy datasets was compared with that of two commonly used similarity measures, namely PCC and the *χ*^2^-statistic, and two established biclustering methods, SAMBA [17] and ISA [14,35]. PCC was chosen as a representative of the global similarity measures used in traditional clustering algorithms, while we included the *χ*^2^-statistic because of its relation to the combinatorial statistic used by ENIGMA (see Algorithm section). The selection of biclustering methods was based on the following criteria: (i) the methods should be non-partitioning in nature, (ii) they should have the capacity to generate overlapping biclusters, (iii) a suitable implementation should be publicly available, and (iv) they should produce a reasonable amount of biclusters (in the order of 10–100) on the modular toy datasets. We used the version of SAMBA [17] incorporated in the EXPANDER 3.0 package [26], and the implementation of ISA [35] available as part of the biclustering tool BicAT [36], both with default parameter settings. The ISA trajectories from randomly chosen starting points (default 100) converge to a limited number of 'fixed point' biclusters. To prune nearly identical modules, we merged ISA biclusters that overlap for more than 80%.

The clustering performance of all methods is only assessed in the gene dimension. Standard internal criteria for partitional clustering performance, such as the silhouette width or Dunn's index [37,38], cannot be used to assess the performance of algorithms that generate overlapping clusters. Instead, we use the *F*-measure and introduce a derivative, the *F'*-measure (also used in the ENIGMA clustering optimization procedure described above), to compare the performance of different clustering methods on artificial datasets. In both cases, the coexpression network generated by a method (either directly or by translating the clusters to network components) is compared to the artificial input coexpression network in terms of true and false positive edges and false negative edges, from which the different flavors of the *F*-measure are calculated (see Additional file 1 Figure S1). The difference between the *F*-measure and the *F'*-measure is that the *F*-measure does not take into account the multiplicity of the inferred edges. In other words, the *F'*-measure penalizes overpredicted (redundant) edges, whereas the *F*-measure does not. This entails that the *F'*-measure is more useful to compare methods that generate overlapping clusters, whereas the *F*-measure can be used more generally to compare methods that generate both overlapping or non-overlapping clusters or pair-wise coexpression networks.

The performance of ENIGMA is tested on two levels by assessing the overlap between the artificial input correlation network and (i) the network of significant correlations obtained in the first step of the ENIGMA algorithm (before clustering, referred to as ENIGMA-N); (ii) the modules inferred by ENIGMA (ENIGMA-M). The output networks for ENIGMA-M and the biclustering methods SAMBA and ISA are obtained by converting the obtained modules/biclusters to fully connected network components. The *χ*^2 ^network is constructed by translating significant *χ*^2 ^correlation *p*-values between the discretized expression profiles to edges in the output network. We used the same discretization threshold (|log_2 _ratio| = 1) and FDR level (0.05) for the *χ*^2 ^and ENIGMA methods. The performance of PCC was measured for different thresholds (for each threshold *t*, gene pairs with PCC > *t *define an edge in the network).

**Figure 2:**
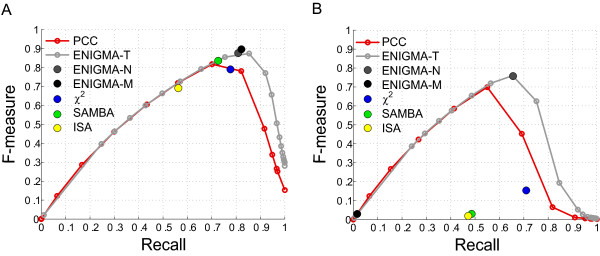
**Performance on artificial data**. Performance of ENIGMA versus other coexpression measures and biclustering methods on (A) modular and (B) non-modular toy datasets. The ENIGMA-T curve shows the performance for the ENIGMA coexpression network at several non-corrected *p*-value thresholds, ENIGMA-N stands for the ENIGMA coexpression network at FDR = 0.05, and ENIGMA-M for the final clustering result.

Using the *F*-measure, ENIGMA outperforms all other methods on the modular artificial data (see Figure 2A and Additional file 1 Tables S1 and S2). The performance of ENIGMA-M was consistently higher than the *χ*^2 ^performance (Δ*F *= 0.11 on average) and the optimal PCC performance (at a PCC threshold of 0.20–0.30 depending on the dataset; Δ*F *= 0.07 on average). The global similarity measure PCC appears to perform surprisingly well. However, the performance of PCC critically depends on the choice of the PCC threshold, and determining the optimal PCC threshold on real data is problematic. In contrast, ENIGMA has the advantage of having an easily tunable significance threshold: the False Discovery Rate (FDR) level. To illustrate this, we plotted the performance curve of ENIGMA for different non-corrected *p*-value thresholds (ENIGMA-T curve), on Figure 2A and 2B. For all artificial datasets, the performance of ENIGMA-N at FDR = 0.05 (medium gray dot) is close to the optimum of this curve, indicating that FDR control at a reasonable level gives near-optimal performance.

Among the biclustering methods, the rather poor performance of the ISA algorithm (Δ*F *with ENIGMA-M = 0.34 on average) may seem somewhat surprising. Prelić et al [18], using the same implementation of ISA but other methods to generate artificial data and to assess biclustering performance, previously established that the performance of ISA decreases with increasing overlap between biclusters. Our results seem to confirm that ISA is not the optimal method in case there is substantial overlap between modules. The performance of ISA did not change significantly when using 500 starting points instead of the default 100 (results not shown).

The performance gain of ENIGMA-M over SAMBA is substantially smaller (Δ*F *= 0.03 on average), and on two out of 10 datasets, the performance of SAMBA was slightly higher than that of ENIGMA-M (see Additional file 1 Tables S1 and S2). A more tangible advantage of ENIGMA over SAMBA (and ISA) is that ENIGMA nearly always recovered the correct number of modules (20 ± 1), whereas SAMBA consistently predicted more modules than there were in the input data (53 ± 6 modules). ISA predicted only one extra module on average, but with a higher variance than ENIGMA (21 ± 4). In other words, SAMBA and to a lesser extent ISA produce more fragmented and/or more redundant modules. Redundancy makes the module output much harder to interpret, but it is not taken into account by the standard *F*-measure.

To quantify the effect of redundancy on the clustering quality, we compared SAMBA, ISA and ENIGMA-M using the *F'*-measure. As in the calculation of the *F'*-measure used in the clustering optimization procedure (see above), edges that are inferred by multiple modules are counted multiple times, but in the present case, multiply defined edges may also occur in the input network if they overlap between multiple artificial input modules (see Additional file 1 Figure S1 B). Specifically, edges that are inferred by *x *output modules and *y *input modules are now counted as *y tp *and *x *- *y fp *in case *x *≥ *y*, or *x tp *and *y *- *x fn *in case *x *<*y*. Using the *F' *criterion, the performance of ENIGMA-M (*F' *= 0.85 ± 0.03) is substantially higher than that of SAMBA (*F' *= 0.74 ± 0.03) and ISA (*F' *= 0.51 ± 0.09, see Additional file 1 Tables S3–S5).

On non-modular artificial data, the performance of ENIGMA-M and the biclustering methods SAMBA and ISA is very low (see Figure 2B and Additional file 1 Tables S6 and S7). This is not surprising since there are no modules to be found in these datasets. In this respect, a particularly attractive feature of ENIGMA is that it finds very few modules in the non-modular data (3 ± 1 modules containing on average 5 genes each, precision of clustering result = 0.27), in contrast to ISA and SAMBA, which recover 78 ± 5 modules (containing on average 27 genes) and 127 ± 2 modules (containing on average 16 genes), respectively. Among the pair-wise methods, ENIGMA-N invariably featured the highest performance, indicating that our combinatorial statistic detects partial coexpression relationships more efficiently than PCC and *χ*^2^. The fact that ENIGMA efficiently uncovers coexpression relationships in non-modular data opens perspectives for the exploration of the less modular parts of expression datasets. Real datasets typically contain a limited number of perturbation experiments that target a few specific processes. These processes can be expected to be rather well resolved in terms of their coexpression relationships, whereas other processes will probably give rise to more fragmented (less modular) regions in the network. Moreover, despite the success of the modularity concept in the analysis of expression data and systems biology in general, it is not inconceivable that transcriptional networks might also contain genuinely non-modular regions.

### Testing on real data: the Rosetta gene expression compendium

#### *p*-value versus log-ratio based discretization

Although useful for testing and comparing methods, artificial datasets do not capture the complexity of real biological systems. Consequently, good performance on artificial data does not guarantee good performance on real biological data. In order to assess the use of ENIGMA for analyzing real data, we applied our methodology to the Rosetta compendium of expression profiles, representing data on 300 different experimental perturbations of *S. cerevisiae *[4]. Experiments on 20 strains that were marked as aneuploid in the original dataset were left out, because they can give rise to artificial expression correlations between genes on the aneuploid chromosomes. The log-ratio expression data and differential expression *p*-values were downloaded in prenormalized and preprocessed form. Genome-wide ChIP data for 102 TFs were obtained from Harbison et al [39]. All genes that are bound with *p *< 0.005 by a certain TF were considered reliable targets. Protein and genetic interactions for *S. cerevisiae *were obtained from the BioGRID database [40].

Using a differential expression *p*-value threshold of 0.01 in the discretization step and an FDR threshold of 0.05 for defining coexpression edges, ENIGMA identified a network of 100,762 significant positive coexpression links and 30,390 negative coexpression links involving 2,871 genes. The clustering parameters (*ν*_1_, *ν*_2_) = (0.30, 0.55) were optimized by MCSA as described in the Algorithm section. To assess the efficiency of the MCSA procedure, we performed an exhaustive screen of the parameter space to locate the global maximum of the *F'*-measure criterion (see Additional file 1 Figure S3). The MCSA procedure found back the global optimum with 100% efficiency.

ENIGMA discovered 206 modules in the Rosetta dataset encompassing 2201 genes and 141 conditions (see supporting data for module details and figures). These numbers seem reasonable given that 130 of the 280 conditions included in the compendium contain less than five differentially expressed genes, which entails that they have a small chance of contributing to a module. Given the low amount of informative conditions, it is not surprising that only a third of the *S. cerevisiae *genes can be included in modules. According to the GO enrichment results, 107 out of 206 modules have a significant degree of functional coherence. Fifty-four modules are enriched in targets of one or more TFs, and 39 modules show enrichment of both GO Biological Process categories and TF binding sites. Together, 60% of the modules show enrichment of GO categories and/or TF binding sites, indicating that our method is capable of identifying biologically relevant expression modules.

In order to qualitatively assess the effect of using a differential expression *p*-value cutoff in the discretization step instead of a fold-change cutoff, we repeated the analysis using a |log_2 _ratio| discretization threshold of 1 (two-fold up- or downregulation). The resulting coexpression network contains 58,612 positive and 2,837 negative links between 2,581 genes. The clustered network contains 206 modules encompassing 1,853 genes. Ninety-three modules exhibit GO enrichment, 47 exhibit TF binding enrichment and 35 exhibit both. Despite the significantly lower amount of connections in the log-ratio network, the number of functionally coherent modules and the number of clustered genes is roughly similar, and the optimized clustering parameters (*ν*_1_, *ν*_2_) = (0.30, 0.55) are identical, indicating that the general structure of the network and its strongest modules are fairly well preserved. Indeed, many highly functionally coherent modules (a.o. related to amino acid metabolism, hexose transport, steroid biosynthesis, iron ion homeostasis, mating) are present in both networks. Not incidentally, many of these modules are related to the processes that were targeted by Hughes et al [4], which can be expected to show a pronounced expression response. However, modules that show less pronounced expression variations, for example the modules related to ribosome biosynthesis, are not recovered in the log-ratio network. This illustrates the main disadvantage of using a fixed log-ratio threshold: different processes show different amplitudes of expression change upon perturbation, which cannot be captured by a single threshold. One could argue that this can easily be remedied by standardizing the expression profiles to zero mean, unit variance before applying the threshold, as is done by some methods, e.g. SAMBA [17]. However, in the case of perturbational data, this manipulation runs the risk of effiectively breaking the connection to the reference condition, thereby distorting the meaning of up- and downregulation and introducing serious artifacts (see Additional file 1 Figure S4).

#### Topological characteristics of the ENIGMA coexpression network

Since many cellular functions are carried out in a highly modular manner [41], most cellular networks, including protein interaction networks, metabolic networks and gene expression networks, are modular in nature [42-47]. On the other hand, many cellular networks, including coexpression networks, have been claimed to exhibit a node degree (*k*) distribution of the power-law type, P(*k*) ~ *k*^-*γ*^, indicative of scale-free properties [47-49]. The coexistence of modularity and a scale-free degree distribution can be explained by assuming a hierarchical modular network organization [43,47,49]. According to this view, the network consists of a hierarchy of nested topological modules of increasing size and decreasing coherence. In other words, small coherent modules combine to form larger and less coherent modules in a hierarchical fashion. At reasonable levels of module resolution, the modules consist mainly of sparsely connected but highly clustered nodes (low *k*, high *C*). The modules are linked together through a small number of highly connected nodes with a low clustering coefficient (high *k*, low *C*), often referred to as hubs. In the case of coexpression networks, these hubs represent genes that are linked to different expression modules depending on the experimental conditions.

**Figure 3:**
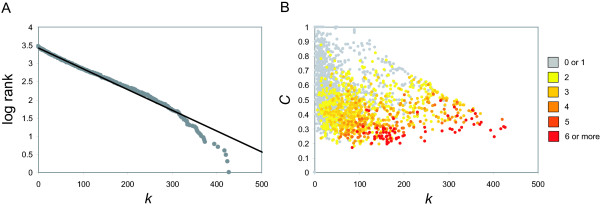
**Topological characteristics of the Rosetta network**. (A) Semilog rank-degree plot for the ENIGMA network inferred from the Rosetta data [4]. (B) Plot of the clustering coefficient of a node's neighborhood as a function of the node degree *k*. The data points are colored according to the number of modules to which the corresponding gene is assigned.

A few papers [50,51] have cast doubt on the ubiquity of power-law degree distributions in biological networks, claiming that some of the supposed power-laws actually turn out to be closer to exponentials when rigorously analyzed. Indeed, the degree distribution of the ENIGMA co-differential expression network appears to be exponentially distributed (Figure 3A), at least for lower *k*. For higher *k*, the picture is different. Relative to the distribution obtained for lower degrees, the most highly connected nodes (hubs) seem to be underconnected. This observation is exactly the opposite of what would be expected for a power-law ('fat-tailed') degree distribution (i.e. highly connected nodes should be overconnected with respect to the exponential distribution), indicating that the coexpression hubs are not nearly as central in the network as would be expected in a scale-free network. However, from the plots of the clustering coefficient *C *versus the degree *k *(Figure 3B), it is apparent that the highly connected nodes still possess hub-like characteristics: they generally have a lower clustering coefficient and are assigned to multiple modules. Thus, highly connected nodes act more as local hubs that hold together a few modules. These hubs, by virtue of their polytomous expression behavior, may represent genes that function at the interface of several processes. An example of genes that probably interface between the cell cycle, mating pheromone response and cell wall biosynthesis is given below. Overall, 1050 genes are linked to 2 or more modules and 115 are linked to 5 or more modules, indicative of extensive crosstalk at the transcriptional level.

#### Comparison between ENIGMA, SAMBA and ISA

Rigorously comparing the performance of (bi)clustering algorithms on real data is extremely difficult, because of the lack of adequate gold standards and the subjectivity of the available external performance criteria [23]. Therefore, we limit ourselves to a more qualitative comparison of the ENIGMA, SAMBA and ISA modules obtained on the Rosetta dataset. SAMBA was run with default parameter settings, for ISA we used the BicAT implementation [36] with parameters *t*_*G *_= 3.1, *t*_*C *_= 2.0 and 10,000 starting points (see [14] for parameter details). The ISA biclusters were pruned by merging biclusters that showed more than 80% overlap. The ISA and SAMBA biclusters were put through the ENIGMA postprocessing pipeline to functionally annotate them and to screen them for TF binding enrichment. SAMBA identified 314 modules containing 3,437 genes and 279 conditions. 203 modules were enriched in one or more GO Biological Process categories, 161 modules were enriched in binding sites for one or more TFs, and 136 modules showed both GO and TF binding enrichment. ISA identified 236 modules containing 3,065 genes and 261 conditions. Eighty-one modules were enriched in one or more GO Biological Process categories, 39 modules were enriched in binding sites for one or more TFs, and 28 modules showed both GO and TF binding enrichment. These numbers are not directly comparable between methods, because of the differing degrees of overlap (redundancy) between modules in the three formalisms. SAMBA generates a lot of biclusters with largely overlapping gene content (but different condition sets), whereas the gene overlap between the ENIGMA modules and especially the pruned ISA modules is more limited. For instance, SAMBA identified 17 modules enriched in conjugation-related genes, containing a total of 46 genes annotated to 'conjugation' in GO (see Table 1). In contrast, ENIGMA and ISA identified fewer conjugation modules (10 and 11, respectively), but containing similar amounts of known conjugation genes (43 and 42, respectively).

Instead of comparing general properties such as the overall coverage of genes and conditions by biclusters, the proportion of GO-enriched modules or the average specificity (functional coherence) of the enriched modules, we focused our comparison on the biological processes that were mainly targeted by Hughes et al [4] (see Table 1), namely mating (conjugation), ergosterol biosynthesis, cell wall biogenesis, oxidative phosphorylation and iron ion homeostasis. All three formalisms uncover modules that are highly enriched for these processes. We used two criteria to assess the module representation of a given GO class *A*, namely the overall recall, or proportion of genes annotated to *A *found across all modules enriched for *A*, and the top module precision, or the proportion of genes in the most significantly enriched module that belong to *A*. SAMBA generally detects slightly more true positive genes than ENIGMA (higher recall), but at the expense of a lower top module precision and a higher amount of modules (see Table 1). ISA generally features a lower recall than SAMBA and ENIGMA, but frequently exhibits better top modules in terms of precision. In short, the main distinction between the formalisms seems to be a difference in balance between precision and recall. Moreover, the interpretation of the criteria defined above is not always straightforward. For instance, a lower top module precision is not always caused by a lack of functional coherence, but may be caused by the presence of genes involved in closely related processes. If we look at the overlap between the gene sets identified by the three methods (see Additional file 1 Figure S5), it becomes clear that all three formalisms add extra information to the global picture. For all 5 processes in Table 1, a sizeable core of genes is identified by all three methods, but the different methods also have substantial idiosyncrasies. For instance, only 25 out of a total of 64 identified conjugation-related genes are found by all three formalisms. Eleven genes are found by ENIGMA and SAMBA but not ISA, two genes are found by ENIGMA and ISA but not SAMBA, and four are shared between SAMBA and ISA but not ENIGMA. Five genes are ENIGMA-specific, 6 are SAMBA-specific and strikingly, 11 are ISA-specific, although ISA identifies the least number of conjugation genes in total and has the 'worst' top module. This illustrates that different methods have different focuses and biases, and that integration of the results of different analysis methods often leads to a better global picture.

**Table 1:** Performance on Rosetta data

						Top module		
						
GO category	# genes	method	# modules	*tp*	*R*	*p*	*tp*	*P*
conjugation (GO:0000746)	117	ENIGMA	10	43	0.37	3.98E-29	23	**0.62**
		SAMBA	17	46	**0.39**	4.10E-29	24	0.55
		ISA	11	42	0.36	1.55E-15	18	0.28
ergosterol biosynthesis (GO:0006696)	26	ENIGMA	4	14	0.54	1.28E-12	9	0.23
		SAMBA	3	16	**0.62**	1.93E-14	15	0.08
		ISA	1	11	0.42	1.23E-19	11	**0.39**
cell wall biogenesis (GO:0042546)	32	ENIGMA	1	8	0.25	2.35E-06	8	0.08
		SAMBA	4	9	**0.28**	6.89E-06	9	0.06
		ISA	1	7	0.22	6.32E-07	7	**0.13**
iron ion homeostasis (GO:0055072)	38	ENIGMA	4	15	0.39	2.35E-16	11	**0.37**
		SAMBA	13	16	**0.42**	3.99E-18	13	0.33
		ISA	2	13	0.34	8.43E-14	13	0.15
oxidative phosphorylation (GO:0006119)	38	ENIGMA	6	23	0.61	3.02E-12	9	0.35
		SAMBA	11	30	**0.79**	2.34E-32	20	**0.44**
		ISA	2	8	0.21	1.20E-05	6	0.14

Comparison of ENIGMA, SAMBA and ISA results for selected biological processes targeted by Hughes et al. [4]. The three middle columns give the number of modules enriched for the GO class in the first column, the total number of genes annotated to that GO class in these modules (*tp*) and the corresponding recall (*R*). The three last columns contain the enrichment *p*-value of the top module, the number of true positives (*tp*) and the proportion of genes in the top module annotated to the respective GO class (precision *P*).

#### Pheromone response modules

**Figure 4:**
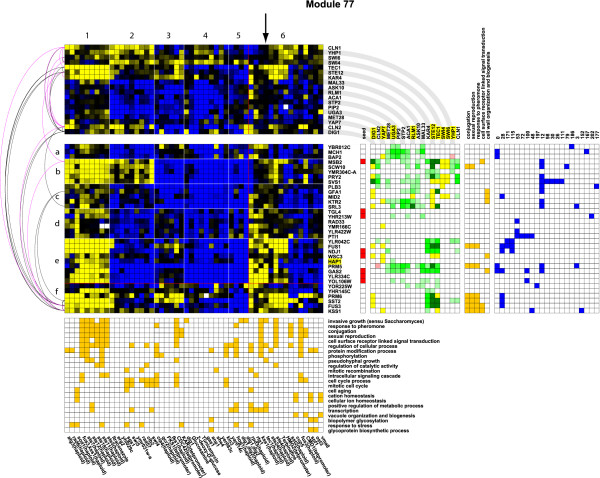
**Mating module 77**. A module enriched in pheromone response genes. The colors of individual spots reflect the expression ratios (experiment vs. control, blue = upregulated, yellow = downregulated, white = missing value). The module is split in leafs in both dimensions based on average linkage clustering using a cos*θ *threshold of 0.65. In order not to crowd the figure, leafs of size < 3 are grouped in a single leaf beyond the red line (rightmost leaf and bottom leaf). Transcription factors are highlighted in yellow in the gene list if there is ChIP data available for them, while other regulators are highlighted in red. To the right of the expression matrix is a column indicating the module's seed genes (red). Further to the right is a matrix depicting the presence of enriched TF binding sites (yellow) and/or significant co- or antiexpression links with potential regulators (green and red, respectively; the hue is proportional to the *p*-value of the link; in case of overlap with an enriched binding site, the field is colored dark green or dark red). The expression profiles of these regulators are depicted on top of the module's expression matrix. Note that regulators that are part of the module are not repeated on top unless they also exhibit significant binding site enrichment. To the far right are matrices depicting the genes' membership of enriched GO categories (orange) and membership of other modules (blue). The black and magenta arcs represent protein and genetic interactions, respectively. The arrow indicates the *tec1*Δ experiment (see main text).

In order to further assess the capacity of ENIGMA to discover biologically relevant connections between genes and processes, we took a closer look at the mating-related ENIGMA modules. The Rosetta compendium contains expression data on at least 20 mating-related perturbations, and consequently the mating pheromone response system is well resolved in the ENIGMA network. Several mating-related modules were uncovered (notably modules 28, 77, 115 and 171, see Figure 1, Figure 4 and supplementary material at [52]).

Module 28 is most strongly related to mating (see Figure 1). Twenty-three of the 37 genes in this module are annotated to the GO category 'conjugation' (GO:0000746, *p *= 3.98E-29). Four TFs exhibit binding enrichment in module 28: Ste12, Dig1, Mcm1 and Tec1. All of these function in the regulation of the mating pheromone response (which includes mating, pseudohyphal and invasive growth). Two regulators show significant coexpression links with the module: Ste12, an important regulator of the mating response (which is in fact part of the module) and Tec1, a transcription factor involved in the regulation of haploid invasive and diploid pseudohyphal growth. The mating and invasive/pseudohyphal growth signaling pathways share many of the same components, and Tec1 is believed to mediate an invasive growth response upon low levels of pheromone signaling [53,54]. Whereas Ste12 appears to be the main regulator for module 28, Tec1 is mainly coexpressed with genes that are shared between module 28 and modules 77, 115 or 171. Modules 115 and 171 are smaller pheromone response-related modules (see figures in supplementary material [52]). Both modules contain Tec1 as a member gene, suggesting that these modules might be more related to pseudohyphal growth than to the conjugation process.

Module 77 exhibits a more complicated substructure, with five major patterns (1–5) in the condition dimension and five in the gene dimension (a-e, leafs 6 and f group smaller leafs, see Figure 4). Most of the known mating-related genes in module 77 reside in the gene leafs e and f. Several genes in these leafs overlap with the mating module 28. In contrast, most of the genes in the leafs b and c overlap with module 12, a module enriched in cell wall biogenesis genes (*p *= 6.26E-10). Nevertheless, most of these genes contain binding sites for Ste12 and Dig1 and some for Tec1, justifying their presence in a pheromone response-related module. While the genes in leaf c appear to be genuinely related to cell wall biogenesis, none of the genes in leaf b is annotated as such. Compared to the genes in leaf c, the genes in leaf b show a distinctive subpattern in condition leaf 2, which mainly contains perturbations that affect the cell cycle, DNA maintenance and DNA repair. Interestingly, the genes in leaf b also distinguish themselves from the ones in leaf c (except for *MID2*) by the presence of TF binding sites for Swi4 and Swi6, which together form the SBF complex that regulates transcription at the G1/S transition [55]. Additionally, the genes in leaf b show strong coexpression links with the cyclins Cln1 and Cln2. Both Swi4 and Swi6 are potential substrates of the protein kinase Cdc28, which is activated by Cln1 and Cln2 [56]. Together, these data suggest that the genes in leaf b function at the interface of cell wall biogenesis, the G1/S transition and mating/filamentous growth. Such a link makes sense since upon activation of the pheromone signaling pathway, the yeast cell cycle is arrested in G1 and extensive cell wall rearrangements take place [57].

Together with the genes in leafs b and f, most genes in leaf e are strongly repressed under the pheromone response-related perturbations in condition leaf 1. Unlike leafs b and f, only a few genes in leaf e (*FUS1 *and *WSC3*) feature bona fide Ste12 or Tec1 binding sites. However, the expression of the other genes in leaf e (with the exception of *HAP1*) is specifically and strongly downregulated upon haploid *TEC1 *deletion (arrow on Figure 4), suggesting that these genes are somehow transcriptionally regulated by Tec1. Further investigation made apparent that several of the genes in leaf e are flanked by or overlapping with an antisense Ty1 retrotransposon long terminal repeat (LTR) on the 3' side (*GAS2*, *YLR334C*, *YOL106W*) or the 5' side (*NDJ1*). The presence of these Ty elements is highly relevant, since *TEC1 *was originally described as a gene required together with *STE12 *for full Ty1 expression [58,59]. Three of these genes (*GAS2*, *YLR334C *and *NDJ1*) were found to be directly or indirectly associated with *TEC1 *in a previous study in which the Rosetta compendium was analyzed using a Bayesian network framework [60]. A peculiar member gene of leaf e is *HAP1*, a transcription factor involved in the regulation of respiratory metabolism in response to levels of heme and oxygen. Interestingly, *HAP1 *also contains a 3' Ty1 insertion in the yeast strain used by Hughes et al (a derivative of strain S288c) [61], which helps explain its puzzling presence in a pheromone response module and strengthens our belief that the Ty1 elements are responsible for the link between leaf e genes and mating genes.

The coexpression of *NDJ1 *with *TEC1 *can be directly explained by the presence of a 5' Ty1 LTR in antisense direction (Ty1 LTRs have been found to drive expression in an orientation-independent manner [59]). For *GAS2*, *YLR334C*, *HAP1 *and *YOL106W*, the situation is different given the 3' location of the flanking Ty1 LTRs. Tec1 and Ste12 activation of these Ty1 elements could in theory cause the production of antisense transcripts of these loci. Since the probes spotted on the microarray used by Hughes et al [4] contained both strands of the gene sequences, such antisense transcripts might be responsible for the observed coexpression pattern.

We did not test the antisense hypothesis; the analysis we present here is merely intended as a use case to show that ENIGMA can generate hypotheses that can be tested in the lab. We did however briefly investigate whether the Ty1-associated genes (or maybe their antisense transcripts) could be functionally related to the mating process. Only two genes in leaf e (*PRM5 *and *FUS1*) are known to be involved in mating. Neither of them is flanked by a Ty1 LTR. One gene overlapping with an antisense Ty1 LTR, *YOL106W*, was previously reported to elicit a mating-related phenotype upon deletion [62]. We performed mating experiments, halo assays and growth assays (see Methods) for two other 3' Ty1-associated genes in leaf e, namely *YLR334C *(overlapping antisense Ty1 LTR) and *GAS2 *(non-overlapping antisense Ty1 LTR), in addition to a wild type (WT) strain and *sst2*Δ, a mutant that is supersensitive to mating factor-induced G1-arrest.

**Figure 5:**
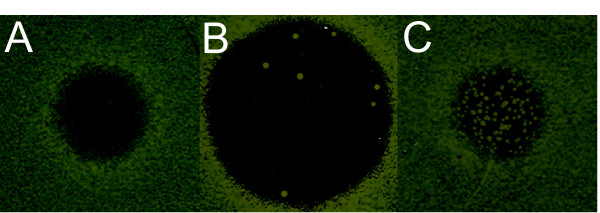
**Halo test for *α*-factor based growth inhibition**. Yeast strains (OD_600_ = 1) were plated on YPD plates and 1000 pmol of *α*-factor was spotted. The pictures are taken after 48 hours of incubation at 30°C. Strains: A: Wild type BY4741 (*MAT***a ***his*3Δ1 *leu*2Δ0 *met*15Δ0 *ura*3Δ0), B: *sst*2Δ, C: *gas*2Δ.

The *ylr334c*Δ deletion strain did not yield an interesting phenotype in any of the assays. The *gas2*Δ deletion strain exhibited an interesting phenotype in the halo assay, characterized by extensive colony formation inside the halo (see Figure 5), which indicates that deletion of *GAS2 *somehow facilitates the recovery from *α*-factor induced growth arrest. In the mating and growth assays, we did not observe any effect of *GAS2 *deletion on the mating ability (see Additional File 1 Table S8, Table S9 and Figure S6). *GAS2 *is homologous to *GAS1*, which encodes a 1,3-*β*-glucanosyltransferase required for cell wall assembly. In a recent study, *GAS2 *was found to be involved in spore wall assembly [63]. Ectopic expression of *GAS2 *under control of the *GAS1 *promoter was found to complement the *gas1*Δ phenotype only partially, and only at pH = 6.5 [63]. It is therefore unlikely that *GAS2 *directly functions in regular cell wall assembly or maintenance. In one hypothetical scenario, antisense transcripts of *GAS2*, produced under control of Tec1, might interfere with the expression of its homolog *GAS1 *and hence indirectly with the formation and maintenance of the cell wall. An altered cell wall morphology might influence the efficiency with which *α*-factor is inactivated, which could explain the observed *gas2*Δ phenotype. Obviously, this is only a hypothesis and much more detailed experimentation is needed to unravel if and how *GAS2 *is linked to the pheromone response pathway. This is however outside the scope of the present study.

### Implementation

ENIGMA is implemented as a command-line Java application that is open-source and freely available (under the GNU General Public License) from [52]. ENIGMA can be used for any organism for which there is sufficient gene expression data available. The only organism-specific part of the ENIGMA algorithm is the functional annotation module, which is based on the BiNGO tool [34]. ENIGMA can be used out-of-the-box for 24 organisms, including yeasts, invertebrates, plants and mammals (see Manual section of [52]). Furthermore, ENIGMA allows the use of custom GO annotation files and GO Consortium files to accommodate other organisms.

## Conclusion

We have developed a novel method, called ENIGMA, to analyze perturbational microarray data. One of the innovations of our methodology is the use of a combinatorial statistic that is capable of detecting significant partial coexpression relationships between genes. In this respect, our method can be considered similar in purpose to biclustering methods, although ENIGMA assesses coexpression links between individual genes rather than expression coherence in a group of genes under a group of conditions. Our method produces both a detailed network of significant pair-wise coexpression links and a high-level representation of the modularity in the expression network.

Tests on artificial data have shown that ENIGMA outperforms other methods, although ENIGMA wins from SAMBA on points rather than by knockout. Similar near-draws with SAMBA were reported earlier for cMonkey [23] and BiMax [18]. This indicates that the (bi)clustering field has matured to a point at which it becomes increasingly difficult to easily improve on the performance of existing methods. However, ENIGMA does have some specific advantages. First, in contrast to other discretization-based methods such as SAMBA, ENIGMA discretizes the expression data based on differential expression *p*-values. Second, ENIGMA efficiently retrieved the correct number of modules from artificial datasets and actively avoids generating redundant modules, which greatly improves the interpretability of the results. Third, ENIGMA's clustering parameters are automatically optimized or can be set on relatively objective grounds. A fourth advantage that is more obvious on real data is the use of ENIGMA's expression module concept for biological discovery. In contrast to the coherent biclusters generated by most methods, an ENIGMA expression module may contain distinctive subpatterns. From our analysis of the Rosetta data, it became apparent that these subpatterns frequently represent more tightly coregulated gene clusters involved in biological processes related to a common functional theme. In our view, the grouping of such different but statistically and functionally connected patterns in one module aids greatly in the biological interpretation of the data and in the assessment of crosstalk between biological processes. The interpretation of a module's substructure is further facilitated by the integration of other data types. This is illustrated in our analysis of module 77, a pheromone response module which shows links to the cell cycle, cell wall biosynthesis and Ty1 LTR-associated genes.

Although numerous approaches have already been used to mine the Rosetta compendium [4,49,60,62,64], ENIGMA offers yet another perspective on the data. This mainly illustrates that the ideal clustering method does not exist [23,65], and that no single approach can extract all the information hidden in large compendium datasets. The elucidation of the regulatory networks governing the many different aspects of cellular function will therefore not only require the integration of different types of data, but also the integrated use of several complementary methods to analyze these data. We believe that ENIGMA constitutes a valuable addition to the existing repertoire of analysis methods.

## Methods

### Mating experiments

Yeast strains were grown overnight in YPD [yeast extract (1%), peptone (2%) and glucose (2%)] and diluted to an OD_600 _= 0.5 in fresh YPD. 500 *μ*l of each strain (*MAT***a**) was mixed with 500 *μ*l of the wild type strain (*MAT****α***). The cells were shaken with 180 rpm at 30°C. At time points 0 h, 4 h and 24 h, 100 *μ*l samples were serially diluted and plated on medium lacking either methionine (*MAT****α***), lysine (*MAT***a**) or methionine and lysine (diploids).

### Halo assay

A halo assay to measure response to and recovery from pheromone-induced growth arrest was performed as follows. Yeast cells (*MAT***a**) were grown overnight and diluted to OD_600 _= 1. 500 *μ*l was plated on YPD plates (1.5% agar in YPD). When the plates were dry, 2 *μ*l of the *α *mating factor (= 1000 pmol) was spotted. The cells were allowed to grow for 48 hrs before the plates were scanned.

### Growth assay

Yeast strains (*MAT***a**) were incubated with the wild type strain (*MAT****α***) for 4 hours as described above and diluted to OD_600 _= 0.1. The length of the lag phase and the maximum growth rate of yeast strains in SDglu without lysine and methionine were monitored automatically by OD_600 _measurements with a BioscreenC apparatus (Labsystems). The parameters were as follows: 300 *μ*l of culture in each well, 30 s of shaking each 3 min (medium intensity), and OD_600 _measurement every hour. Readings are saturated at OD_600_s above 1.5.

## Authors' contributions

SM designed the study, developed the methods, analyzed and interpreted the data, and wrote the paper. PVD performed and analyzed the mating experiments, and MK designed the study and supervised the project.

## Supplementary Material

Additional file 1 The supplementary pdf file accompanying this article contains the Supplementary Methods, Tables S1–S9 and Figures S1–S6. Additional supplementary material, including test datasets and module figures, can be downloaded from [52]. Click here for file
