# Supplementary material for: Extracting expression modules from perturbational gene expression compendia
Source: BMC Syst Biol. 2008 Apr 10;2:33. doi: 10.1186/1752-0509-2-33 (PMC2386865; doi:10.1186/1752-0509-2-33)
Supplement: Additional file 1 — The supplementary pdf file accompanying this article contains the Supplementary Methods, Tables S1–S9 and Figures S1–S6. Additional supplementary material, including test datasets and module figures, can be downloaded from [52]. [file 1752-0509-2-33-S1.pdf]

Supplementary material: Extracting expression modules from perturbational gene expression compendia

Steven Maere*[1,2], Patrick Van Dijck[3,4] , Martin Kuiper[1,2]

[1]Department of Plant Systems Biology, VIB, Technologiepark 927, B-9052 Ghent, Belgium
[2]Department of Molecular Genetics, Ghent University, Technologiepark 927, B-9052 Ghent, Belgium
[3]Department of Molecular Microbiology, VIB, Kasteelpark Arenberg 31, B-3001 Leuven, Belgium
[4]Laboratory of Molecular Cell Biology, Katholieke Universiteit Leuven, Kasteelpark Arenberg 31, B-3001 Leuven, Belgium

Email: Steven Maere - steven.maere@psb.ugent.be; Patrick Van Dijck - patrick.vandijck@bio.kuleuven.be; Martin Kuiper - martin.kuiper@psb.ugent.be;

*Corresponding author

Abstract

Background: Compendia of gene expression profiles under chemical and genetic perturbations constitute an invaluable resource from a systems biology perspective. However, the perturbational nature of such data imposes specific challenges on the computational methods used to analyze them. In particular, traditional clustering algorithms have difficulties in handling one of the prominent features of perturbational compendia, namely partial coexpression relationships between genes. Biclustering methods on the other hand are specifically designed to capture such partial coexpression patterns, but they show a variety of other drawbacks. For instance, some biclustering methods are less suited to identify overlapping biclusters, while others generate highly redundant biclusters. Also, none of the existing biclustering tools takes advantage of the staple of perturbational expression data analysis: the identification of differentially expressed genes.

Results: We introduce a novel method, called ENIGMA, that addresses some of these issues. ENIGMA leverages differential expression analysis results to extract expression modules from perturbational gene expression data. The core parameters of the ENIGMA clustering procedure are automatically optimized to reduce the redundancy between modules. In contrast to biclusters produced by other methods, ENIGMA modules may show internal substructure, i.e. subsets of genes with distinct but significantly related expression patterns. The grouping of these (often functionally) related patterns in one module greatly aids in the biological interpretation of the data. We show that ENIGMA outperforms other methods on artificial datasets, using a quality criterion that, unlike other criteria, can be used for algorithms that generate overlapping clusters and that can be modified to take redundancy between clusters into account. Finally, we apply ENIGMA to the Rosetta compendium of expression profiles for *Saccharomyces cerevisiae* and we analyze one pheromone response-related module in more detail, demonstrating the potential of ENIGMA to generate detailed predictions.

Conclusions: It is increasingly recognized that perturbational expression compendia are essential to identify the gene networks underlying cellular function, and efforts to build these for different organisms are currently underway. We show that ENIGMA constitutes a valuable addition to the repertoire of methods to analyze such data.

Supplementary methods

Combinatorial distribution

Consider the expression profiles of two genes A and B under N perturbations. Each gene is represented by a profile of N fields. Experiments in which the gene is up- or downregulated relative to the control condition are labelled blue resp. yellow. The remaining fields are left black. In order to compare the profiles of the two genes A and B, let us assume that profiles A and B have a_x and b_x blue fields respectively, as well as a_y and b_y yellow fields, and that they have x blue and y yellow fields in common. The probability $P(x, y)$ that the profiles A and B overlap on exactly x blue and y yellow positions when randomly distributing the colored positions on both profiles is given by Equation (1) in the article. This equation can be rewritten as:

$$\sum_{x'=x}^{\min(a_x,b_x)} \sum_{y'=y}^{\min(a_y,b_y)} \binom{x'}{x}\binom{y'}{y} P(x', y') = \frac{\binom{a_x}{x}\binom{a_y}{y}\binom{N-x-y}{b_x-x}\binom{N-b_x-y}{b_y-y}}{\binom{N}{b_x}\binom{N-b_x}{b_y}} \quad \text{(S1)}$$

Assume that profile A is given, and that we consider all possible profiles B with b_x blue and b_y yellow positions. The denominator of the term on the right hand side represents the total number of possible profiles B. The numerator represents the number of ways that x blue and y yellow matching positions can be picked, while the residual positions are chosen at random. All profiles B having at least x blue and y yellow matches are counted at least once. But, for a shuffled profile B with $x' > x$ blue and y yellow matches, for example, one can choose the x matching positions in the numerator of the right hand side in $C(x', x)$ different ways. In other words, this profile will be counted for each of these $C(x', x)$ choices of x matching positions. In general, a profile having $x' \geq x$ blue and $y' \geq y$ yellow matches will be counted $C(x', x).C(y', y)$ times, hence the summation on the left hand side of Equation S1.

Equation S1 is the equation for three profile 'colors' (blue, yellow and black). It can be extrapolated to higher or lower dimensionality. The two-color formula reads:

$$\sum_{x'=x}^{a_x} \binom{x'}{x} P(x') = \frac{\binom{a_x}{x}\binom{N-x}{b_x-x}}{\binom{N}{b_x}} \quad \text{(S2)}$$

While we were unable to find a closed-form solution for $P(x, y)$, there is a simple solution for $P(x)$. The two-color (say blue and yellow) problem can be formulated as follows: when randomly picking b_x blue positions out of N (i.e. when constructing profile B), what is the probability that x of them belong to the class of a_x blue positions on profile A? Since this represents a random selection (without repetition) among objects of two distinct types (match and no match), $P(x)$ should be equivalent to the hypergeometric distribution. Hence :

$$\sum_{x'=x}^{a_x} \binom{x'}{x} \frac{\binom{a_x}{x'}\binom{N-a_x}{b_x-x'}}{\binom{N}{b_x}} = \frac{\binom{a_x}{x}\binom{N-x}{b_x-x}}{\binom{N}{b_x}} \quad \text{(S3)}$$

Indeed, this combinatorial identity can be proven using the WZ method [1]. The WZ method, due to H.S. Wilf and D. Zeilberger, is a 'proof machine', or automated method, to certify the truth of combinatorial identities. The method returns a rational function $R(x, x')$, the proof certificate, which can be used to rigorously reconstruct the proof of a combinatorial identity. The proof certificate for Equation S3 is given below:

$$R(x, x') = \frac{(-x' + x)(N - a_x - b_x + x')}{(-b_x + x)(-a_x + x)} \quad \text{(S4)}$$

References

1. Petkovsek M, Wilf H, Zeilberger D: *A=B*. A K Peters, Ltd., Wellesley (MA) 1997.

Supplementary Tables and Figures

	PCC (max)	χ^2 (FDR=0.05)	ENIGMA-N (FDR=0.05)	ISA	SAMBA	ENIGMA-M
1	0.6692	0.7437	0.7563	0.5117	0.6799	0.7546
2	0.6785	0.7425	0.7549	0.3618	0.6715	0.7645
3	0.6834	0.7131	0.7837	0.3313	0.8122	0.8167
4	0.7586	0.6942	0.7588	0.2359	0.6928	0.7851
5	0.6866	0.7332	0.7398	0.2921	0.7235	0.7455
6	0.6394	0.6917	0.6735	0.4195	0.7685	0.6978
7	0.7877	0.6652	0.7269	0.3524	0.7376	0.7431
8	0.6432	0.7059	0.7226	0.3837	0.7800	0.7457
9	0.6393	0.7295	0.7583	0.3974	0.7346	0.7786
10	0.7014	0.7767	0.8087	0.5646	0.7264	0.8217

Table S1: Recall of different methods when applied to 10 modular artificial datasets. PCC (max) indicates the maximum performance PCC threshold (generally PCC=0.2 or PCC=0.3). ENIGMA-N stands for the coexpression network produced in the first stage of ENIGMA, while ENIGMA-M stands for the final clustering result.

	PCC (max)	χ^2 (FDR=0.05)	ENIGMA-N (FDR=0.05)	ISA	SAMBA	ENIGMA-M
1	0.7797	0.7351	0.8264	0.5972	0.7788	0.8233
2	0.7988	0.7596	0.8457	0.5115	0.7811	0.8611
3	0.8062	0.7724	0.8678	0.4896	0.8917	0.8985
4	0.7785	0.7575	0.8488	0.3672	0.8131	0.8788
5	0.8039	0.7353	0.8485	0.4081	0.8231	0.8542
6	0.7676	0.6933	0.7954	0.5671	0.8571	0.8218
7	0.7780	0.7450	0.8311	0.5005	0.8430	0.8506
8	0.7745	0.7346	0.8256	0.5413	0.8621	0.8518
9	0.7745	0.7579	0.8539	0.5521	0.8439	0.8742
10	0.8177	0.7903	0.8763	0.6923	0.8355	0.8965

Table S2: F-measure of different methods when applied to 10 modular artificial datasets.

	ISA	SAMBA	ENIGMA-M
1	0.6651	0.6841	0.8810
2	0.8617	0.7950	0.9843
3	0.9378	0.7421	0.9983
4	0.7952	0.7203	0.9978
5	0.6696	0.7528	1.0000
6	0.8713	0.8176	0.9992
7	0.8285	0.8265	0.9936
8	0.9157	0.6962	0.9926
9	0.9023	0.7431	0.9965
10	0.8765	0.7104	0.9842

Table S3: P' (adjusted precision, see Figure S1) of (bi)clustering methods when applied to 10 modular artificial datasets.

	ISA	SAMBA	ENIGMA-M
1	0.5020	0.6782	0.7425
2	0.3532	0.6632	0.7527
3	0.3217	0.8040	0.8030
4	0.2260	0.6797	0.7672
5	0.2853	0.7175	0.7325
6	0.4112	0.7605	0.6871
7	0.3419	0.7268	0.7266
8	0.3736	0.7756	0.7337
9	0.3864	0.7238	0.7632
10	0.5533	0.7197	0.8113

Table S4: R' (adjusted recall, see Figure S1) of (bi)clustering methods when applied to 10 modular artificial datasets.

	ISA	SAMBA	ENIGMA-M
1	0.5721	0.6812	0.8059
2	0.5011	0.7232	0.8531
3	0.4791	0.7718	0.8901
4	0.3520	0.6994	0.8674
5	0.4002	0.7347	0.8456
6	0.5587	0.7881	0.8143
7	0.4840	0.7734	0.8394
8	0.5307	0.7338	0.8437
9	0.5394	0.7333	0.8644
10	0.6784	0.7150	0.8894

Table S5: F'-measure (adjusted F-measure, see Figure S1) of (bi)clustering methods when applied to 10 modular artificial datasets.

	PCC (max)	χ^2 (FDR=0.05)	ENIGMA-N (FDR=0.05)	ISA	SAMBA	ENIGMA-M
1	0.5150	0.6413	0.5932	0.4689	0.5230	0.0100
2	0.4910	0.6573	0.6132	0.4509	0.4810	0.0080
3	0.5340	0.6800	0.6200	0.5060	0.5080	0.0120
4	0.5140	0.6580	0.6080	0.4540	0.4780	0.0060
5	0.4870	0.6212	0.5731	0.4589	0.5050	0.0120
6	0.5360	0.6720	0.6120	0.5320	0.5020	0.0200
7	0.4860	0.6440	0.5920	0.4320	0.4820	0.0140
8	0.5280	0.6880	0.6360	0.4300	0.4760	0.0040
9	0.5500	0.7100	0.6560	0.4720	0.4860	0.0160
10	0.5130	0.6453	0.6032	0.4569	0.5030	0.0240

Table S6: Recall of different methods when applied to 10 non-modular artificial datasets.

	PCC (max)	χ^2 (FDR=0.05)	ENIGMA-N (FDR=0.05)	ISA	SAMBA	ENIGMA-M
1	0.6675	0.1356	0.7193	0.0189	0.0274	0.0195
2	0.6499	0.1435	0.7321	0.0166	0.0285	0.0154
3	0.6881	0.1336	0.7355	0.0190	0.0291	0.0231
4	0.6667	0.1367	0.7343	0.0179	0.0278	0.0118
5	0.6471	0.1235	0.7018	0.0177	0.0291	0.0228
6	0.6872	0.1345	0.7260	0.0180	0.0280	0.0377
7	0.6420	0.1168	0.7133	0.0174	0.0273	0.0267
8	0.6795	0.1388	0.7509	0.0175	0.0298	0.0079
9	0.6989	0.1537	0.7575	0.0187	0.0297	0.0294
10	0.6632	0.1282	0.7227	0.0167	0.0255	0.0436

Table S7: F-measure of different methods when applied to 10 non-modular artificial datasets.

	lys-			met-			met-lys-		
	0h	4h	24h	0h	4h	24h	0h	4h	24h
WT	9	97	570	9	127	400	0	9	320
*ylr334c*Δ	13	92	700	14	93	750	0	2	300
*ylr452c*Δ	9	31	40	11	122	1080	0	1	10
*ylr343w*Δ	13	139	440	14	138	390	0	15	230

Table S8: Cell counts (x 10^4) for haploid *MAT***a** (lys-) and *MAT*$\boldsymbol{\alpha}$ (met-) cells and diploid cells (met-lys-) in 100 μl culture samples after 0h, 4h and 24h.

	diploid/*MAT***a**			diploid/*MAT*$\boldsymbol{\alpha}$			*MAT***a**/*MAT*$\boldsymbol{\alpha}$		
	0h	4h	24h	0h	4h	24h	0h	4h	24h
WT	0	9.3	56.1	0	7.1	80	100	76.4	142.5
*ylr334c*Δ	0	2.2	42.9	0	2.2	40	92.9	98.9	93.3
*ylr452c*Δ	0	3.2	25	0	0.8	0.9	81.8	25.4	3.7
*ylr343w*Δ	0	10.8	52.3	0	10.9	59	92.9	100.7	112.8

Table S9: Percentages of diploid cells relative to *MAT***a** and *MAT*$\boldsymbol{\alpha}$ cells, and of *MAT***a** cells relative to *MAT*$\boldsymbol{\alpha}$ cells after 0h, 4h and 24h.

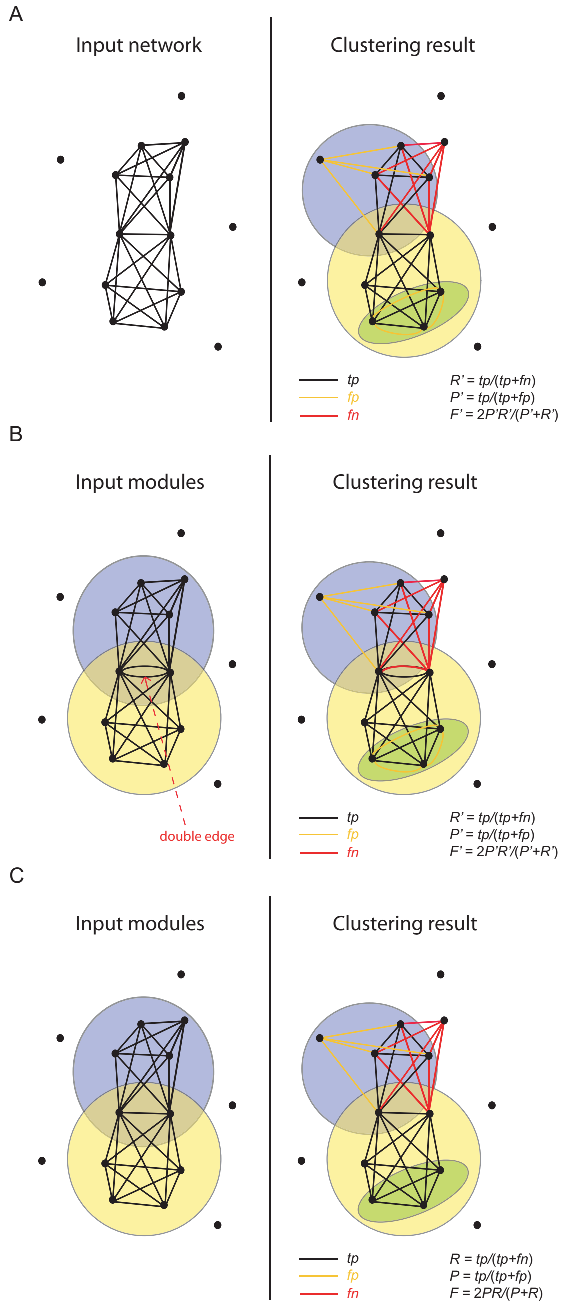

Figure S1: Graphical depiction of the calculation of the F- and F'-measures for comparing a clustering output to known input networks or clusters. (A) The F'-measure used in the optimization of ENIGMA's clustering parameters. Edges that are inferred by multiple (x) output modules are counted as 1 tp and $x-1$ fp, so that redundancy in the output clusters (e.g. the green cluster in the output) is penalized by an increase in the false positive (fp) count. Lack of overlap between clusters gives rise to an increase in the false negative (fn) count. (B) F'-measure in case the input modules are known (e.g. from artificial data). In this case, edges shared by multiple input modules are also counted multiple times. Edges that are inferred by x output modules and y input modules are now counted as y tp and $x-y$ fp in case $x \geq y$, or x tp and $y-x$ fn in case $x < y$. When the input and output clusterings are identical, the F'-measure equals 1. (C) The standard F-measure does not penalize overpredicted edges.

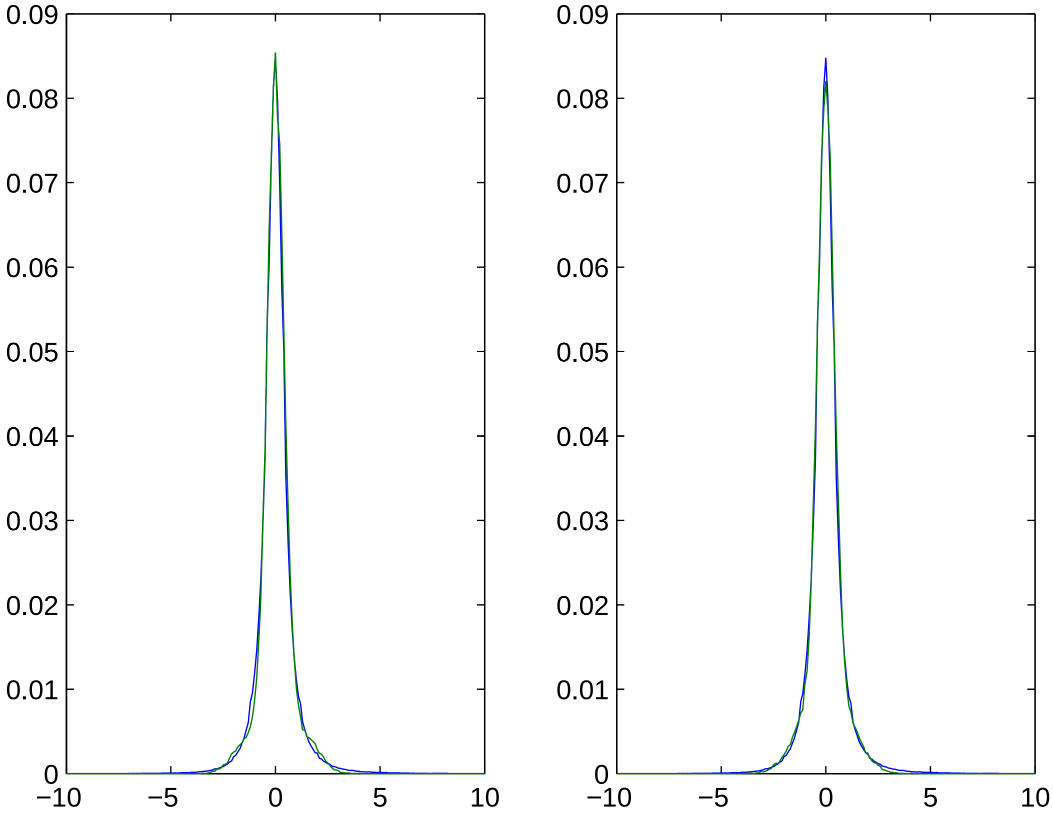

Figure S2: Distribution of the $\log_2$ ratio expression values in the Rosetta dataset, multiplied by a scale factor of 3.3 (blue) compared to the modular (A) and non-modular (B) artificial data distribution (green).

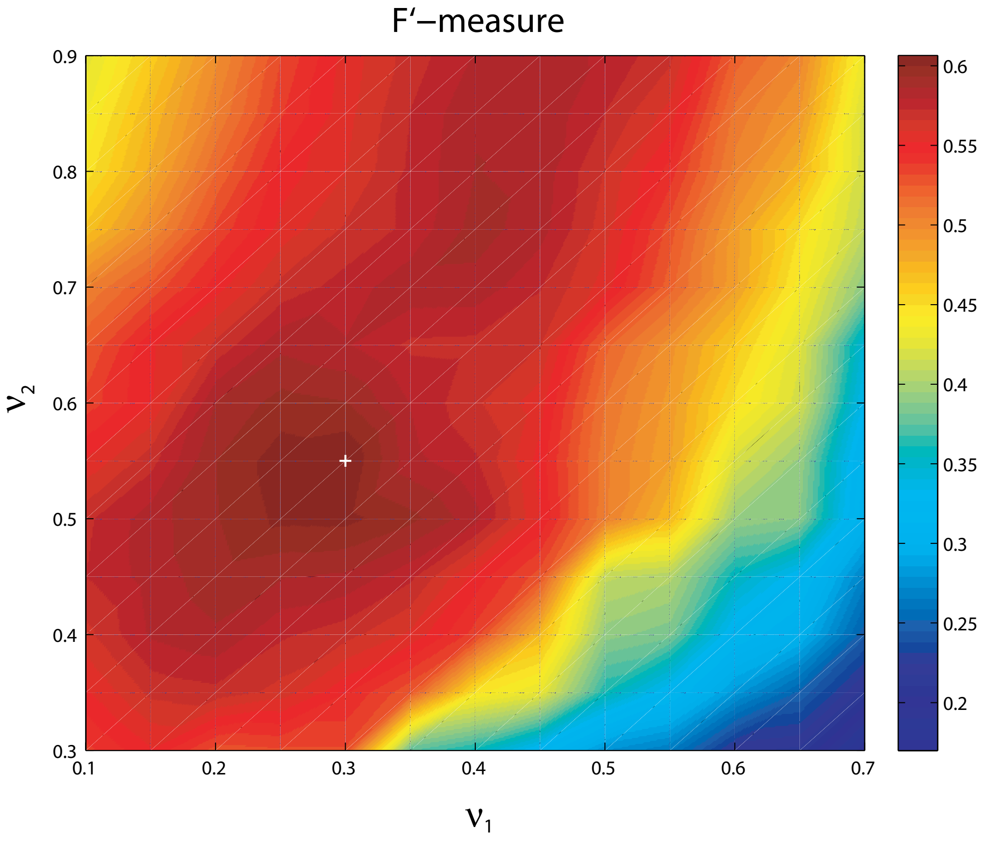

Figure S3: F'-measure of the ENIGMA clustering result on the Rosetta dataset, for the relevant range of possible values for the clustering parameters ν_1 and ν_2. The F'-measure is used to assess the quality with which the modules cover the coexpression graph obtained in the first stage of the ENIGMA algorithm. The white cross at $(\nu_1, \nu_2) = (0.30, 0.55)$ indicates the maximum of the F'-measure surface (at parameter precision 0.01). The simulated annealing strategy built into ENIGMA finds this maximum with 100% efficiency.

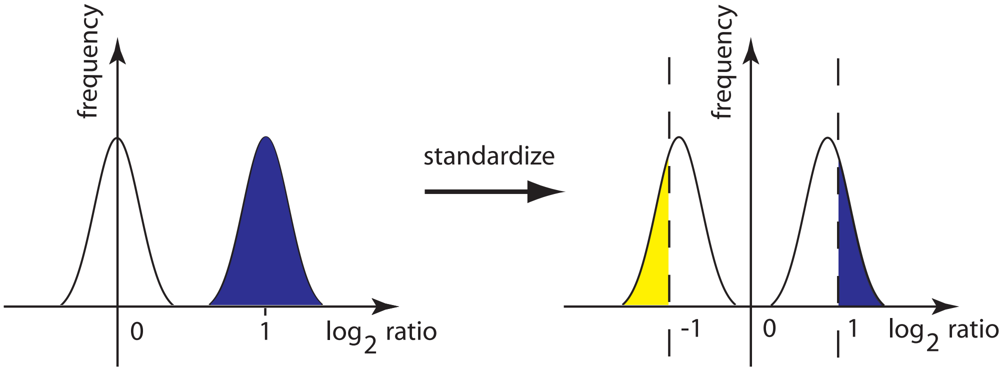

Figure S4: Illustration of the possible effect of expression profile standardization on discretization into categories of up- and downregulation and unchanged expression. Shown on the left is the log ratio expression value distribution of a hypothetical gene whose expression is unchanged in half of the conditions (white), and upregulated in the other half (blue). After standardization of the expression profile to zero mean and unit variance and discretization, the gene appears to be downregulated in part of the 'unchanged expression' conditions, and upregulated in only part of the 'upregulated expression' conditions (the drawing is only approximate).

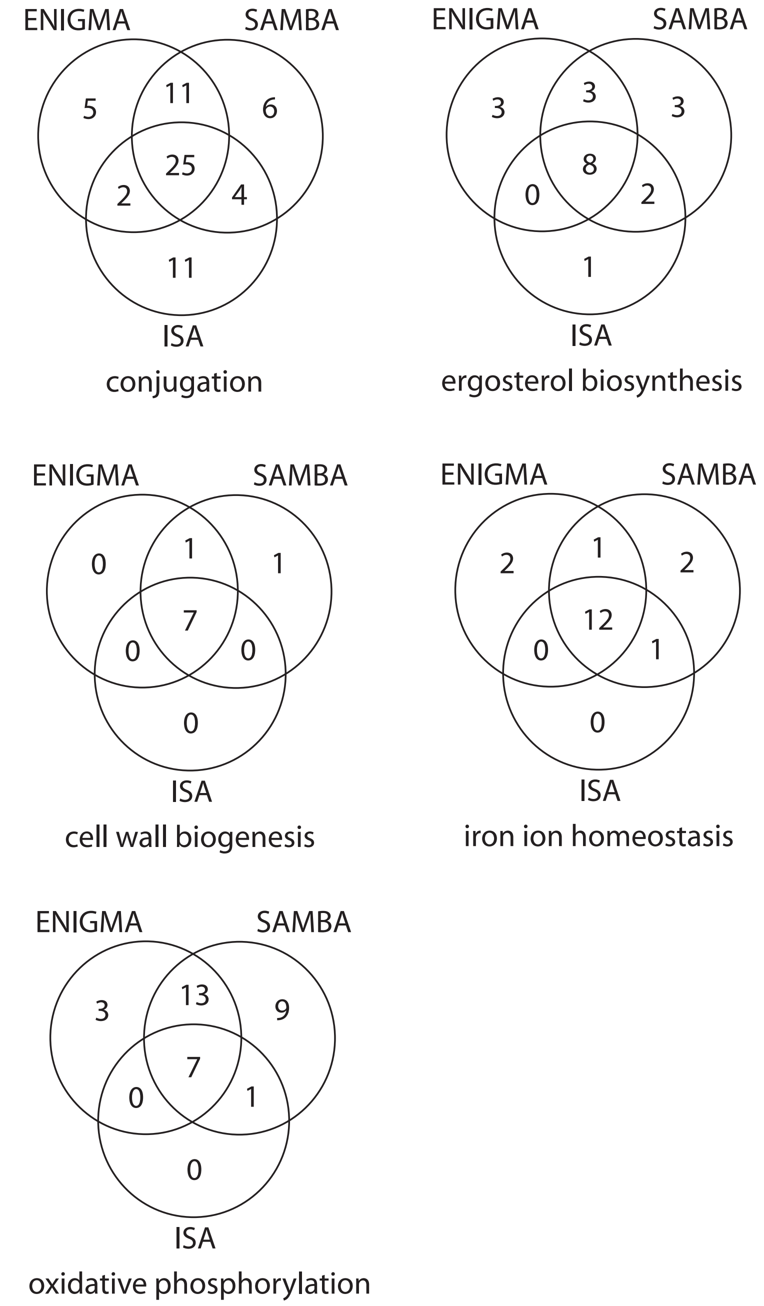

Figure S5: Overlap between the 'true positive' genes recovered in enriched SAMBA, ISA and ENIGMA modules, for selected processes targeted in the Rosetta compendium

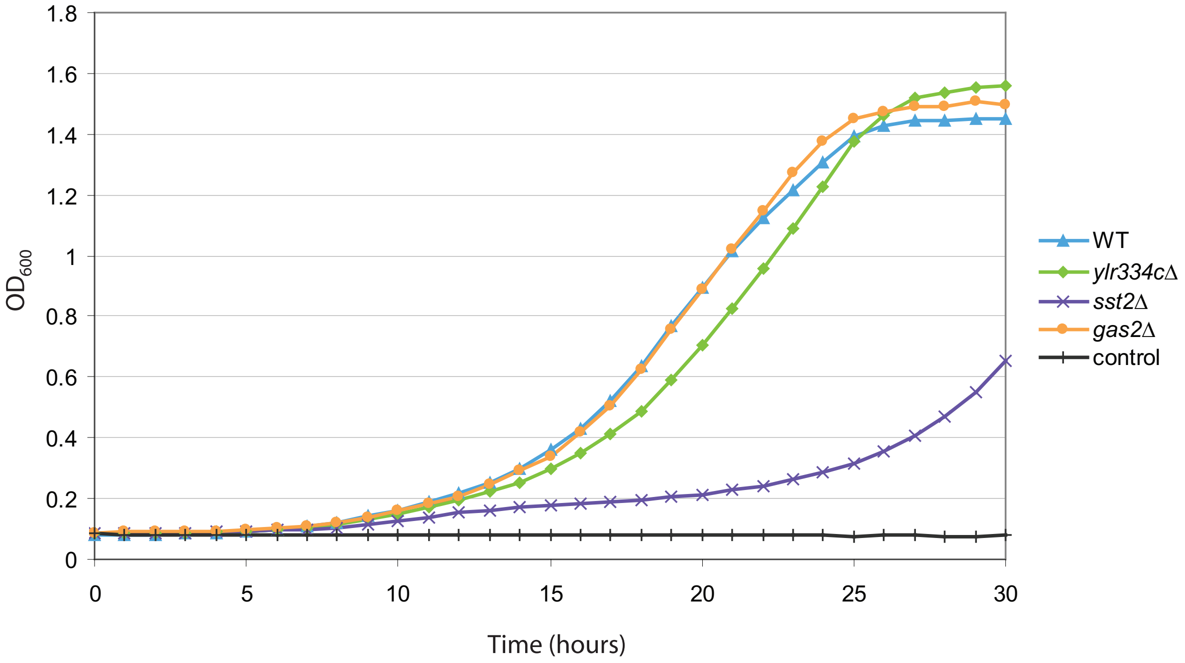

Figure S6: Growth curves for the tested yeast strains in SDglu without lysine and methionine, after 4 hours of mating.
